# Mammographic density and risk of breast cancer by age and tumor characteristics

**DOI:** 10.1186/bcr3570

**Published:** 2013-11-04

**Authors:** Kimberly A Bertrand, Rulla M Tamimi, Christopher G Scott, Matthew R Jensen, V Shane Pankratz, Daniel Visscher, Aaron Norman, Fergus Couch, John Shepherd, Bo Fan, Yunn-Yi Chen, Lin Ma, Andrew H Beck, Steven R Cummings, Karla Kerlikowske, Celine M Vachon

**Affiliations:** 1 Channing Division of Network Medicine, Department of Medicine, Brigham and Women’s Hospital and Harvard Medical School, 181 Longwood Ave, Boston, MA 02115, USA; 2 Department of Epidemiology, Harvard School of Public Health, 677 Huntington Avenue, Boston, MA 02115, USA; 3 Division of Biomedical Statistics and Informatics, Mayo Clinic College of Medicine, 200 First Street SW, Rochester, MN 55905, USA; 4 Department of Anatomic Pathology, Mayo Clinic College of Medicine, 200 First Street SW, Rochester, MN 55905, USA; 5 Department of Health Sciences Research, Division of Epidemiology, Mayo Clinic College of Medicine, 200 First Street SW, Rochester, MN 55905, USA; 6 Division of Experimental Pathology, Department of Laboratory Medicine and Pathology, Mayo Clinic College of Medicine, 200 First Street SW, Rochester, MN 55905, USA; 7 Department of Radiology, University of California, 1 Irving Street, AC109San Francisco, CA 94143, USA; 8 Department of Pathology, University of California, 505 Parnassus Avenue, Room M559 Box 0102, San Francisco, CA 94143, USA; 9 Department of Medicine, University of California, Box 1793, 1635 Divisadero St. Suite 600, San Francisco, CA, USA; 10 Department of Pathology, Beth Israel Deaconess Medical Center and Harvard Medical School, 330 Brookline Avenue, Boston, MA 02215, USA; 11 San Francisco Coordinating Center, California Pacific Medical Center Research Institute, 475 Brannan Street, Suite 220, San Francisco, CA 94107, USA; 12 Departments of Medicine and Epidemiology and Biostatistics, University of California, 4150 Clement Street, Mailing Code 111A1, San Francisco, CA 94121, USA; 13 General Internal Medicine Section, Department of Veterans Affairs, University of California, 4150 Clement St, Mailing Code 111A1, San Francisco, CA 94121, USA

## Abstract

**Introduction:**

Understanding whether mammographic density (MD) is associated with all breast tumor subtypes and whether the strength of association varies by age is important for utilizing MD in risk models.

**Methods:**

Data were pooled from six studies including 3414 women with breast cancer and 7199 without who underwent screening mammography. Percent MD was assessed from digitized film-screen mammograms using a computer-assisted threshold technique. We used polytomous logistic regression to calculate breast cancer odds according to tumor type, histopathological characteristics, and receptor (estrogen receptor (ER), progesterone receptor (PR), human epidermal growth factor receptor (HER2)) status by age (<55, 55–64, and ≥65 years).

**Results:**

MD was positively associated with risk of invasive tumors across all ages, with a two-fold increased risk for high (>51%) versus average density (11-25%). Women ages <55 years with high MD had stronger increased risk of ductal carcinoma *in situ* (DCIS) compared to women ages 55–64 and ≥65 years (*P*_age-interaction_ = 0.02). Among all ages, MD had a stronger association with large (>2.1 cm) versus small tumors and positive versus negative lymph node status (*P*’s < 0.01). For women ages <55 years, there was a stronger association of MD with ER-negative breast cancer than ER-positive tumors compared to women ages 55–64 and ≥65 years (*P*_age-interaction_ = 0.04). MD was positively associated with both HER2-negative and HER2-positive tumors within each age group.

**Conclusion:**

MD is strongly associated with all breast cancer subtypes, but particularly tumors of large size and positive lymph nodes across all ages, and ER-negative status among women ages <55 years, suggesting high MD may play an important role in tumor aggressiveness, especially in younger women.

## Introduction

Mammographic density (MD) is one of the strongest risk factors for breast cancer: women in the highest quartile of MD have four to six times increased risk of breast cancer compared with those in the lowest quartile [1]. The magnitude of risk associated with established breast cancer risk factors, such as age, menopausal status, and parity, differs according to tumor characteristics [2-6], suggesting breast cancer is a heterogeneous disease that develops through different pathways and may have etiologically distinct tumor subtypes. Because MD is a potential risk factor for prediction models, understanding whether MD is a risk factor for all breast cancer subtypes, including the most aggressive, and whether these associations are consistent at all ages is highly significant.

To date, the MD and breast cancer association according to different tumor characteristics is inconsistent [7-20]. Some previous analyses report no clear differences in strength of association by tumor characteristics [10-12,16-20], while others suggest the association between MD and breast cancer risk differs by estrogen receptor (ER) status [13-16], invasiveness [15,21], and tumor size [7-9]. Most [7-9,15,22], but not all [10], prior studies have reported stronger associations of MD with large tumors versus small tumors, which could reflect delays in diagnosis due to reduced sensitivity of mammography [23] and/or aggressive tumor biology.

Given these conflicting results and known differences in the tumor biology of younger and older women diagnosed with breast cancer [24-26], we conducted a large, comprehensive pooled analysis of six case–control studies, two of which were nested within cohorts, to examine risk of breast cancer associated with MD by age and tumor characteristics.

## Methods

### Study populations

Participating studies included the Mayo Mammography Health Study (MMHS) [27,28], the Mayo Clinic Breast Cancer Study (MCBCS) [29,30], the Nurses’ Health Study (NHS) and NHSII [31-33], the Mayo Clinic Mammography Study (MCMAM) [34], and the San Francisco Bay Area Breast Cancer SPORE and San Francisco Mammography Registry (SFMR) [35-37] (Table 1; see also Additional file 1). From all studies, we excluded breast cancer cases diagnosed within 6 months of mammography and their matched controls, to minimize prevalent cancers at the time of mammography. Covariate information was obtained from medical record review (MCMAM), self-administered questionnaires (NHS, NHSII), or both (MMHS, MCBCS), prior to mammography (NHS and NHSII) or at the time of mammography (MCBCS, MMHS, MCMAM, and SFMR). In total, these analyses included 3,414 breast cancer cases and 7,199 controls.

**Table 1:** Characteristics of the study populations

**Study name (abbreviation)**	**Reference**	**Study design**	**Number of cases/controls**	**Enrollment year(s)**	**Mammogram film view**	**Median interval (years) between mammogram and diagnosis (index date)**	**Source of cases**	**Source of pathology**	**Source of covariate data**
Mayo Mammography Health Study (MMHS)	[27,28]	Nested case–control study	404/1,207	2003 to 2006	CC average	3.6	Linkage to clinic and state cancer registries	Clinic and three state cancer registries;medical records	Questionnaire and medical record review (BMI)
Mayo Clinic Breast Cancer Study (MCBCS)	[29,30]	Case–control study	261/179	2001 to 2008	CC contralateral	1.3	Clinic	Clinic cancer registry; medical records	Questionnaire and medical record review (BMI)
Nurses’ Health Study (NHS)	[31,33]	Nested case–control study	1,108/2,163	1989 to 1990	CC average	5.2	Self-report	Pathology reports and tumor tissue microarray	Questionnaire
Nurses’ Health Study II (NHSII)	[32]	Nested case–control study	365/992	1996 to 1999	CC average	4.2	Self-report	Pathology reports and tumor tissue microarray	Questionnaire
Mayo Clinic Mammography Study (MCMAM)	[34]	Case–control study	372/679	1997 to 2001	CC average	7.1	Clinic	Clinic cancer registries; medical records	Medical record review
Bay Area Breast Cancer SPORE and San Francisco Mammography Registry (SFMR)	[35-37]	Nested case–control study	904/1,979	1996 to 2007	CC contralateral	3.1	Linkage to state-wide SEER program	SEER	Questionnaire

BMI, body mass index; CC, craniocaudal mammogram view; SEER, Surveillance, Epidemiology and End Results.

This study was approved by the Institutional Review Boards at the Mayo Clinic, Brigham and Women’s Hospital, the University of California, San Francisco (UCSF), and the Connecticut Department of Public Health Human Investigations Committee. Informed consent was obtained or implied by return of questionnaires (NHS, NHSII).

### Assessment of mammographic density

MD was measured using two computer-assisted threshold techniques (Cumulus [38] and UCSF custom mammographic density software [39]) from digitized images of prediagnostic film screening mammograms of the craniocaudal view. With the exception of NHS and NHSII, for which the average percent MD of both breasts was used, MD was estimated from the contralateral breast for cases and the corresponding side for matched controls. Previous studies have documented that similar results are obtained from an average of both breasts and from a randomly selected side [40] and the correlation between the right and left breast MD is 0.96 [41].

Because of known differences in the distribution of percent MD between readers [42,43], we standardized MD measurements made within each study (Additional file 1) for pooled analyses. Briefly, we focused on women without breast cancer and estimated study-specific linear age trends in the medians of the logit-transformed MD values using quantile regression. We removed the study-specific age trends by computing the difference between each individual’s observed logit-transformed MD and the age-predicted median logit-transformed MD from the corresponding study set. We standardized the variability across studies by dividing the residuals within each study by their corresponding interquartile range, and multiplied these rescaled residual values by the interquartile range of the original residuals from all studies. This ensured that the variability in standardized logit-transformed MD was consistent across studies, and was roughly equivalent to the observed variability in logit-transformed MD. Finally, we estimated an overall age by logit-transformed MD trend from the original data, and added the age-predicted median logit-transformed MD to the rescaled residuals from each individual. This incorporated the known age trend in MD into the standardized logit-transformed MD measurements. These logit-transformed MD values were back-transformed to the original scale for use in analyses.

### Assessment of tumor characteristics among cases

Information on tumor type, histology, grade, nodal involvement, tumor size, and ER, progesterone receptor (PR), and human epidermal growth factor receptor 2 (HER2) status was obtained from state-wide Surveillance Epidemiology and End Results programs (SFMR), pathology reports (NHS and NHSII), state and clinic cancer registries (MMHS, MCMAM, MCBCS), and medical records (MMHS, MCMAM, MCBCS). For 66 cases in NHS or NHSII with missing receptor data on pathology reports, receptor status was obtained from immunohistochemical staining performed on paraffin sections of the tumor tissue microarray according to a standard protocol [44]. A large proportion of cases (39%) were missing HER2 status; and 31% of cases were diagnosed prior to the availability of herceptin and the need for HER2 testing, primarily from the MCMAM and NHS studies that were mostly postmenopausal cases. Another 2% were cases with borderline HER2 results (2+ without available fluorescent in situ hybridization) and were not used in analyses.

### Statistical analyses

Standardized percent MD was categorized as 0 to 10%, 11 to 25%, 26 to 50%, and 51%+, with the average MD group (11 to 25%) as the reference group consistent with previous analyses [17,37]. We fit polytomous (multinomial) logistic regression models to estimate odds ratios (ORs) and 95% confidence intervals for the associations of MD with overall breast cancer as well as with breast cancer defined by tumor type (invasive or ductal carcinoma *in situ* (DCIS)), histologic type (ductal or lobular), grade (well differentiated, moderately differentiated, or poorly differentiated), tumor size (<1.1 cm, 1.1 to 2.0 cm, or 2.1+ cm), involvement of lymph nodes (positive or negative), and receptor status (ER-positive or ER-negative, PR-positive or PR-negative, HER2-positive or HER2-negative).

We pooled data across the six studies and adjusted for study, age (continuous), and body mass index (continuous) in multivariable models. We further considered potential confounding by parity (nulliparous, parous, or unknown) and first-degree family history of breast cancer (yes, no, or unknown) by evaluating the magnitude of change in ORs observed after including each potential confounder individually in the model. Postmenopausal hormone therapy (current estrogen alone, current estrogen plus progesterone, never/former, or unknown) was also evaluated as a confounder among postmenopausal women in the subset of studies for which this information was available (MMHS, NHS, NHSII, and SFMR). Addition of these variables to the models did not substantially change risk estimates and they were not included in final models. Results were similar in sensitivity analyses restricted to Caucasians only (data not shown).

Because prior studies suggest differences in the strength of the association of MD with breast cancer by age [45], and since tumor biology varies with age [24-26], analyses were conducted across all women and stratified by three age groups (<55 years, 55 to 64 years, and ≥65 years) to best capture these differences. We evaluated whether the associations between MD and breast cancer differed by specific tumor characteristics, both overall and within age group, using polytomous logistic regression models (*P*_heterogeneity_). For subtypes with a natural ordering, including tumor size and grade, tests of trend (*P*_trend_) across categories were used to assess significance. Formal tests of interaction (*P*_age-interaction_) assessed the significance of differential MD associations with each of the breast cancer characteristics and subtypes across the three age groups.

Prior to pooling data across the six studies, study-specific estimates were obtained by fitting separate models for each study and assessing individual associations between MD and each tumor subtype. We assessed the statistical significance of differences in associations by study through testing for interactions between study group and MD category in the pooled analysis, and found no evidence of differences across study (all *P* values >0.29).

Analyses were performed using SAS software (version 9.3; SAS Institute, Cary, NC, USA). All statistical tests were two-sided and *P* <0.05 was considered statistically significant.

## Results

Overall, the mean age at mammogram was 57 years among both cases and controls. The mean time to diagnosis was 4.5 years (range: 0.5 to 17.6). Among both cases and controls, the mean percent MD decreased with age, but in each age group the mean percent MD was higher among cases versus controls (Table 2). DCIS was more common among younger women compared with older women, while invasive breast cancers in women <55 years at mammography generally displayed more aggressive tumor characteristics compared with women ≥55 years (Table 3).

**Table 2:** Baseline characteristics of study population by age

	**Age <55 years**		**Age 55 to 64 years**		**Age ≥65 years**	
	**Cases**	**Controls**	**Cases**	**Controls**	**Cases**	**Controls**
*N*	1,533	3,373	1,021	2,031	860	1,795
Percent mammographic density	40.7 (19.6)	33.6 (20.2)	29.2 (18.0)	23.7 (17.6)	24.8 (17.1)	21.1 (16.2)
Age at mammogram	47.3 (4.5)	47.4 (4.4)	59.4 (2.9)	59.4 (2.9)	71.0 (5.2)	71.2 (5.1)
Age at diagnosis	51.8 (5.6)	–	63.8 (4.1)	–	75.0 (5.5)	–
Body mass index	24.4 (6.5)	25.4 (6.3)	26.1 (7.1)	26.2 (5.6)	25.6 (8.5)	26 (5.6)
Body mass index categories (kg/m^2^)						
<25	845 (55.1%)	1,902 (56.4%)	438 (42.9%)	964 (47.5%)	322 (37.4%)	849 (47.3%)
25 to 29	432 (28.2%)	831 (24.6%)	332 (32.5%)	642 (31.6%)	262 (30.5%)	602 (33.5%)
30 to 34	130 (8.5%)	352 (10.4%)	138 (13.5%)	275 (13.5%)	151 (17.6%)	221 (12.3%)
35+	76 (5.0%)	249 (7.4%)	84 (8.2%)	140 (6.9%)	71 (8.3%)	110 (6.1%)
Unknown	50 (3.3%)	39 (1.2%)	29 (2.8%)	10 (0.5%)	54 (6.3%)	13 (0.7%)
Menopausal status						
Premenopausal	948 (61.8%)	2,163 (64.1%)	16 (1.6%)	44 (2.2%)	0 (0%)	0 (0%)
Postmenopausal	473 (30.9%)	1,032 (30.6%)	1,001 (98.0%)	1,979 (97.4%)	860 (100%)	1794 (99.9%)
Unknown	112 (7.3%)	178 (5.3%)	4 (0.4%)	8 (0.4%)	0 (0%)	1 (0.1%)
Parity						
Nulliparous	294 (19.2%)	638 (18.9%)	130 (12.7%)	249 (12.3%)	114 (13.3%)	235 (13.1%)
Parous	1,145 (74.7%)	2,652 (78.6%)	824 (80.7%)	1,686 (83.0%)	674 (78.4%)	1,428 (79.6%)
Unknown	94 (6.1%)	83 (2.5%)	67 (6.6%)	96 (4.7%)	72 (8.4%)	132 (7.4%)
Postmenopausal hormone therapy^a^						
Not current user	158 (40.9%)	411 (45.7%)	404 (48.6%)	1030 (58.6%)	361 (57.8%)	1,010 (69.1%)
Current, estrogen	81 (21.0%)	233 (25.9%)	144 (17.3%)	311 (17.7%)	100 (16.0%)	199 (13.6%)
Current, estrogen + progestin	126 (32.6%)	222 (24.7%)	215 (25.9%)	308 (17.5%)	84 (13.4%)	124 (8.5%)
Unknown	21 (5.4%)	34 (3.8%)	68 (8.2%)	108 (6.1%)	80 (12.8%)	129 (8.8%)
Family history						
No	1,207 (78.7%)	2,970 (88.1%)	777 (76.1%)	1,719 (84.6%)	627 (72.9%)	1,450 (80.8%)
Yes	256 (16.7%)	384 (11.4%)	210 (20.6%)	292 (14.4%)	190 (22.1%)	332 (18.5%)
Unknown	70 (4.6%)	19 (0.6%)	34 (3.3%)	20 (1.0%)	43 (5.0%)	13 (0.7%)

Data presented as mean (standard deviation) or *n* (%). ^a^Among postmenopausal women in the Mayo Mammography Health Study, the Nurses’ Health Study, the Nurses’ Health Study II, and the San Francisco Bay Area Breast Cancer SPORE and San Francisco Mammography Registry.

**Table 3:** Distribution of breast cancer cases from six studies by age and tumor characteristics

**Tumor type**	**Age <55 years**		**Age 55 to 64 years**		**Age ≥65 years**	
	***N***	**%**	***N***	**%**	***N***	**%**
Total controls	3,373	68.8	2,031	66.5	1,795	67.6
Total cases	1,533	31.2	1,021	33.5	860	32.4
Invasive	1,227	80.0	858	84.0	760	88.4
*In situ*	295	19.2	160	15.7	100	11.6
Unknown	11	0.7	3	0.3	0	0.0
**Tumor characteristics**						
Histology						
Ductal	988	80.5	654	76.2	545	71.7
Lobular	123	10.0	110	12.8	114	15.0
Mixed	65	5.3	55	6.4	51	6.7
Unknown/Other	51	4.2	39	4.6	50	6.6
Histologic grade						
Well differentiated	292	23.8	248	28.9	259	34.1
Moderately differentiated	466	38.0	320	37.3	295	38.8
Poorly differentiated	342	27.9	165	19.2	134	17.6
Unknown	127	10.4	125	14.6	72	9.5
Tumor size						
0.1 to 1.0 cm	378	30.8	312	36.7	279	36.7
1.1 to 2.0 cm	496	40.4	330	39.3	299	39.3
2.1+ cm	310	25.3	184	20.7	157	20.7
Unknown	43	3.5	32	3.3	25	3.3
Involvement of lymph nodes						
Negative	810	66.0	610	71.1	500	65.8
Positive	347	28.3	192	22.4	148	19.5
Unknown	70	5.7	56	6.5	112	14.7
Estrogen receptor status						
Negative	228	18.6	139	16.2	82	10.8
Positive	949	77.3	674	78.6	644	84.7
Borderline/Unknown	50	4.1	45	5.2	34	4.5
Progesterone receptor status						
Negative	329	26.8	232	27.0	157	20.7
Positive	844	68.8	580	67.6	566	74.5
Borderline/Unknown	54	4.4	46	5.4	37	4.9
HER2 status						
Negative	674	54.9	359	41.8	383	50.4
Positive	142	11.6	63	7.3	65	8.6
Borderline/Unknown	411	33.5	436	50.8	312	41.1

HER2, human epidermal growth factor receptor 2.

### Overall and invasive breast cancer and ductal carcinoma *in situ*

**Figure 1:**
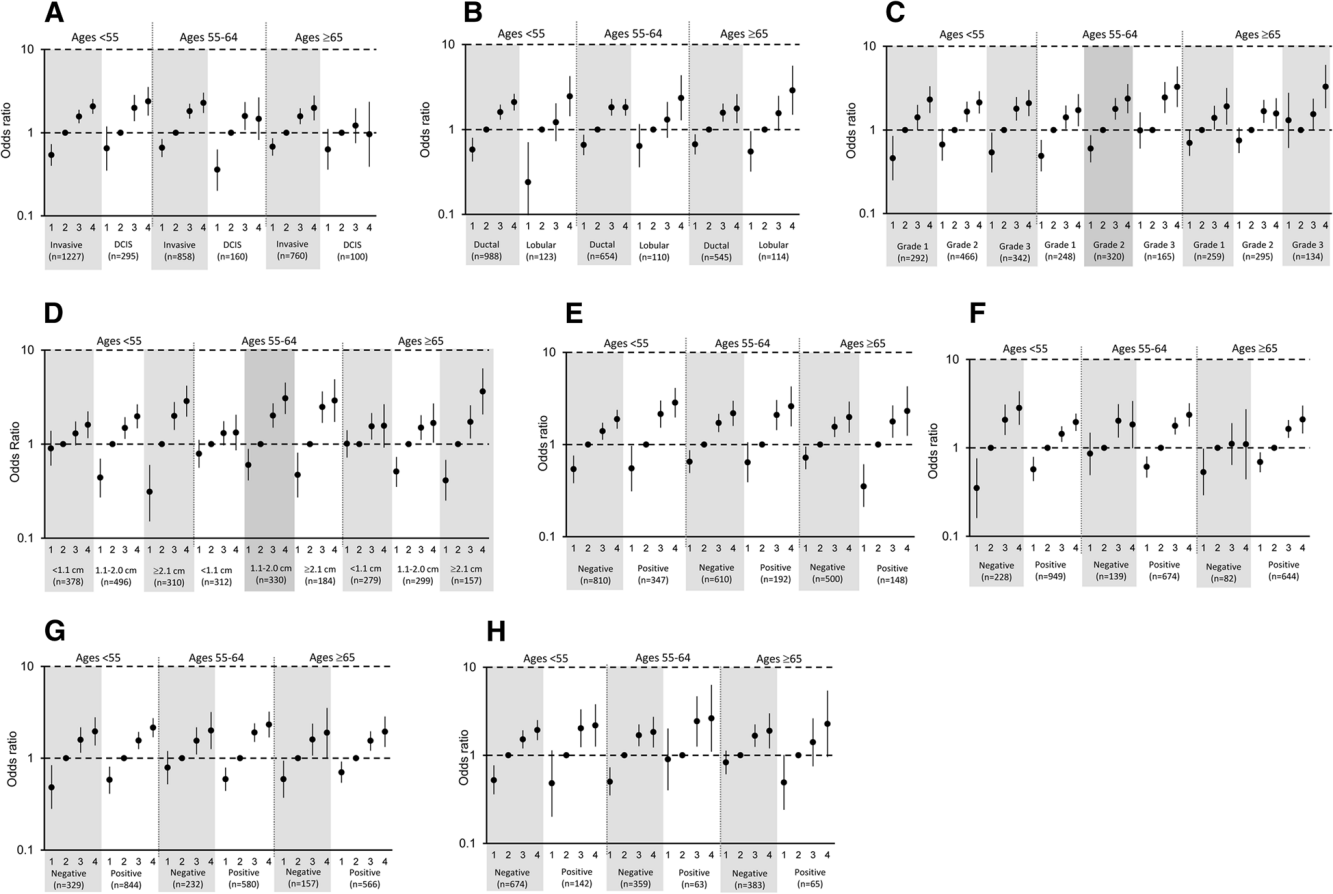
**Pooled associations of categorical mammographic density for tumor type and morphological subtypes of invasive breast cancer by age.** Odds ratios and 95% confidence intervals adjusted for age, body mass index, and study are shown for each category of mammographic density (MD): 1, 0 to 10% MD; 2, 11 to 25% MD (reference); 3, 26 to 50% MD; 4, 51% + MD). **(A)** Tumor type. **(B)** Histology. **(C)** Tumor grade. **(D)** Tumor size. **(E)** Lymph node involvement. **(F)** Estrogen receptor (ER) status. **(G)** Progesterone receptor (PR) status. **(H)** Human epidermal growth factor receptor 2 (HER2) status. DCIS, ductal carcinoma *in situ.*

A positive association of MD with breast cancer overall, adjusted for age and body mass index, was seen for each study (Table S1 in Additional file 2). In the pooled analysis, MD was positively associated with breast cancer risk overall and with invasive cancer, after adjusting for study, body mass index, and age, with similar magnitudes of effect in each MD category (Table 4). However, the difference in the magnitude of association of MD with breast cancer by tumor type (invasive vs. DCIS) varied significantly across age groups (*P*_age-interaction_ = 0.02; Table S2 in Additional file 2). There were similar associations of MD with invasive breast cancer across age groups (Figure 1; see also Table S2 in Additional file 2). But the associations of MD with DCIS differed, primarily for women aged <55 years compared with those aged ≥55 years. Younger women with high MD had increased risk of DCIS (ORs for MD 26 to 50% and 51% + vs. MD 11 to 25% were 1.99 and 2.39, respectively) that was not as strong in older age groups (corresponding ORs of 1.59 and 1.47 for age 55 to 64, and 1.22 and 0.96 for age ≥65 years) (Figure 1; see also Table S2 in Additional file 2).

**Table 4:** Associations of categorical mammographic density with breast cancer overall and by morphological subtypes

	**Number of cases**	**Number of controls**	**Odds ratio (95% CI)**^**a**^	***P***_**heterogeneity**_^**b**^
Overall breast cancer				
0 to 10%	430	1,485	0.65 (0.56, 0.74)	
11 to 25% (reference)	872	2,248	1.00 (reference)	
26 to 50%	1,379	2,386	1.65 (1.48, 1.84)	
51%+	733	1,080	2.15 (1.88, 2.46)	
**Invasiveness**				
Invasive				0.46
0 to 10%	372	1,485	0.66 (0.57, 0.76)	
11 to 25% (reference)	730	2,248	1.00 (reference)	
26 to 50%	1,129	2,386	1.65 (1.47, 1.86)	
51%+	605	1,080	2.21 (1.92, 2.55)	
*In situ*				
0 to 10%	52	1,485	0.54 (0.38, 0.75)	
11 to 25% (reference)	139	2,248	1.00 (reference)	
26 to 50%	240	2,386	1.60 (1.28, 2.00)	
51%+	124	1,080	1.87 (1.42, 2.48)	
**Histology**^**c**^				0.04
Ductal				
0 to 10%	281	1,485	0.66 (0.56, 0.78)	
11 to 25% (reference)	556	2,248	1.00 (reference)	
26 to 50%	884	2,386	1.66 (1.46, 1.88)	
51%+	466	1,080	2.14 (1.83, 2.51)	
Lobular				
0 to 10%	44	1,485	0.54 (0.37, 0.78)	
11 to 25% (reference)	100	2,248	1.00 (reference)	
26 to 50%	119	2,386	1.36 (1.03, 1.80)	
51%+	84	1,080	2.69 (1.93, 3.74)	
**Histologic grade**				0.89
Well differentiated				
0 to 10%	107	1,485	0.61 (0.47, 0.77)	
11 to 25% (reference)	228	2,248	1.00 (reference)	
26 to 50%	295	2,386	1.41 (1.17, 1.70)	
51%+	169	1,080	2.12 (1.68, 2.67)	
Moderately differentiated				
0 to 10%	146	1,485	0.69 (0.56, 0.86)	
11 to 25% (reference)	270	2,248	1.00 (reference)	
26 to 50%	430	2,386	1.70 (1.44, 2.02)	
51%+	232	1,080	2.31 (1.87, 2.84)	
Poorly differentiated				
0 to 10%	75	1,485	0.65 (0.48, 0.87)	
11 to 25% (reference)	148	2,248	1.00 (reference)	
26 to 50%	273	2,386	1.90 (1.53, 2.35)	
51%+	143	1,080	2.34 (1.80, 3.04)	
**Tumor size**				<0.001
<1.1 cm				
0 to 10%	179	1,485	0.93 (0.76, 1.14)	
11 to 25% (reference)	265	2,248	1.00 (reference)	
26 to 50%	353	2,386	1.37 (1.15, 1.63)	
51%+	172	1,080	1.61 (1.29, 2.01)	
1.1 to 2.0 cm				
0 to 10%	121	1,485	0.54 (0.43, 0.67)	
11 to 25% (reference)	294	2,248	1.00 (reference)	
26 to 50%	453	2,386	1.64 (1.39, 1.93)	
51%+	253	1,080	2.27 (1.86, 2.77)	
2.1+ cm				
0 to 10%	56	1,485	0.42 (0.30, 0.57)	
11 to 25% (reference)	154	2,248	1.00 (reference)	
26 to 50%	280	2,386	2.07 (1.67, 2.57)	
51%+	159	1,080	3.12 (2.41, 4.04)	
**Involvement of lymph nodes**				0.01
Negative				
0 to 10%	257	1,485	0.67 (0.57, 0.79)	
11 to 25% (reference)	508	2,248	1.00 (reference)	
26 to 50%	744	2,386	1.54 (1.35, 1.76)	
51%+	406	1,080	2.07 (1.76, 2.44)	
Positive				
0 to 10%	65	1,485	0.52 (0.38, 0.70)	
11 to 25% (reference)	157	2,248	1.00 (reference)	
26 to 50%	298	2,386	1.99 (1.61, 2.45)	
51%+	164	1,080	2.67 (2.07, 3.44)	
**Estrogen receptor status**				0.66
Negative				
0 to 10%	48	1,485	0.61 (0.43, 0.88)	
11 to 25% (reference)	106	2,248	1.00 (reference)	
26 to 50%	192	2,386	1.79 (1.39, 2.30)	
51%+	102	1,080	2.18 (1.61, 2.96)	
Positive				
0 to 10%	305	1,485	0.65 (0.56, 0.77)	
11 to 25% (reference)	596	2,248	1.00 (reference)	
26 to 50%	880	2,386	1.60 (1.41, 1.82)	
51%+	479	1,080	2.22 (1.9, 2.60)	
**Progesterone receptor status**				0.10
Negative				
0 to 10%	90	1,485	0.65 (0.50, 0.85)	
11 to 25% (reference)	187	2,248	1.00 (reference)	
26 to 50%	288	2,386	1.59 (1.30, 1.94)	
51%+	151	1,080	1.99 (1.56, 2.55)	
Positive				
0 to 10%	265	1,485	0.66 (0.56, 0.78)	
11 to 25% (reference)	512	2,248	1.00 (reference)	
26 to 50%	782	2,386	1.65 (1.45, 1.89)	
51%+	424	1,080	2.28 (1.93, 2.68)	
**HER2 status**				0.69
Negative				
0 to 10%	189	1,485	0.66 (0.54, 0.80)	
11 to 25% (reference)	367	2,248	1.00 (reference)	
26 to 50%	560	2,386	1.63 (1.40, 1.90)	
51%+	298	1,080	2.06 (1.70, 2.49)	
Positive				
0 to 10%	31	1,485	0.62 (0.40, 0.97)	
11 to 25% (reference)	62	2,248	1.00 (reference)	
26 to 50%	116	2,386	1.94 (1.40, 2.69)	
51%+	60	1,080	2.33 (1.57, 3.45)	

CI, confidence interval; HER2, human epidermal growth factor receptor 2. ^a^Adjusted for age, body mass index, and study.

^b^Test of heterogeneity in associations by subtype (*P*_trend_ for categories with natural ordering; that is, tumor size and histologic grade).

^c^Mixed and other histology categories are excluded.

### Grade, invasive histology, size and nodal status

MD was similarly associated with breast cancers of various grades (Table 4). Further, this association did not differ when stratified by age group (*P*_age-interaction_ =0.21) (Figure 1; see also Table S3 in Additional file 2). MD was also similarly associated with both ductal and lobular histology among all ages combined (Table 4). Despite evidence of heterogeneity in the association by histologic subtype overall (*P*_heterogeneity_ = 0.04), the magnitude of the association was similar within each age group (all *P*_heterogeneity_ ≥0.12) and there was no evidence of significant interaction by age (*P*_age-interaction_ = 0.15). Also, higher MD was positively associated with invasive tumors of all sizes but the association was stronger for large tumors (that is, 2.1+ cm) compared with small tumors <1.1 cm among all women combined (*P*_trend_ <0.01; Table 4) and within age groups (Figure 1; see also Table S3 in Additional file 2). The ORs for MD 51% + versus MD 11 to 25% ranged from 1.33 to 1.61 for invasive tumors <1.1 cm and from 2.88 to 3.65 for tumors 2.1+ cm. Low MD (that is, 0 to 10%) was not significantly associated with a reduced risk of tumors <1.1 cm compared with the referent category MD 11 to 25%; however, for tumors >1.1 cm, low MD was significantly associated with lower risk (ORs: 0.31 to 0.60) (Figure 1; see also Table S3 in Additional file 2). MD was associated with both lymph node-positive and lymph node-negative tumors among all ages combined, with a stronger association of MD with node-positive than node-negative tumors (*P*_heterogeneity_ <0.01) (Table 4). The MD association with breast cancer defined by nodal status was not different by age group (*P*_age-interaction_ = 0.24), although there was evidence of a stronger association for node-positive versus node-negative tumors among women ages ≥65 (*P*_heterogeneity_ = 0.04).

### Estrogen receptor, progesterone receptor and HER2 status

Associations between MD and breast cancer defined by ER status differed by age group (*P*_age-interaction_ = 0.04). For women aged <55 years, a stronger association was observed for ER-negative disease (OR: 2.84, 95% confidence interval: 1.83, 4.40) versus ER-positive disease (OR: 1.96; 95% confidence interval: 1.56, 2.45) for MD 51% + versus MD 11 to 25% (*P*_heterogeneity_ = 0.09), while associations for women aged ≥55 years were nonstatistically significantly stronger for ER-positive tumors versus ER-negative tumors. MD was similarly associated with PR-negative and PR-positive tumors in all age groups (*P*_age-interaction_ = 0.10). While there was evidence of a significant interaction between MD and breast cancers by HER2 status and age group (*P*_**a**ge-interaction_ = 0.03), MD was positively associated with both HER2-negative and HER-positive disease in all age groups and there were no clear patterns of differences in associations (Figure 1; see also Table S3 in Additional file 2).

## Discussion

In this large pooled analysis we confirmed a positive association between MD and breast cancer risk. Women with high MD (51%+) had an approximately two-fold higher risk of breast cancer compared with women with MD 11 to 25%, and those with less extensive mammographic density (0 to 10%) had almost one-half the risk as those with 11 to 25% MD. This translates to a relative risk of around four comparing the highest category of MD with the lowest, similar to prior studies. Our findings suggest that MD is a risk factor for all histologic/morphologic subtypes of breast cancer examined, but a stronger risk factor for tumors of large (vs. small) size and node-positive (vs. node-negative) disease among all women. We found differential associations of MD and breast cancer defined by tumor type (DCIS vs. invasive cancer), ER status and HER2 status, by age group (<55 years, 55 to 64 years and ≥65 years).

Most previous studies of the association between MD and breast cancer tumor characteristics have focused on hormone receptor status, with somewhat conflicting results [9-11,13-17,20,37,46,47] – in part because most studies have not examined associations by age group and/or menopausal status. Several studies, including a recent meta-analysis [19], reported no difference in associations between MD and breast cancer by ER status [9,10,19,20,37,46,47], while others reported stronger associations for ER-positive disease [13,14,18]. In contrast, a significantly stronger effect of MD on postmenopausal breast cancer was reported for ER-negative compared with ER-positive tumors among the NHS cohort, but the number of ER-negative cases was small [15]. Findings from the current pooled analysis stratified by age group, which included women in the NHS [15], suggest that MD is more strongly associated with ER-negative breast cancer (vs. ER-positive) among women <55 years old compared with women aged 55 years and older. Like other studies [37], we found no difference in associations by PR status; however, one recent case-only study found that tumors diagnosed in women with denser breasts were more likely to be PR-positive compared with tumors in women with more fatty breasts [18]. Our findings of MD positively associated with both HER2-negative and HER2-positive tumors within age groups are consistent with prior studies [11,15,19,20], including a meta-analysis of studies that did not show MD differentially associated by tumors defined by HER2 across all ages combined [19]. The significant interaction between MD, HER2 status, and age that we observed may have been influenced by small numbers of HER2-positive tumors in older age groups and/or due to chance. In addition, HER2 status was unknown for a moderate portion of cases, with the primary reason being a diagnosis date prior to when HER2 was clinically tested. Since the two older studies were comprised of postmenopausal cases, it is not surprising that we found those without HER2 more likely to be older and slightly more likely to be node-negative (80% of cases with unknown/borderline HER2 status were node-negative vs. 70% of those with known HER2 status). Otherwise, the majority of tumor characteristics were similar between the two groups. The results with HER2 will need to be confirmed in other studies.

Among women aged <55 years who had high MD, we noted similar associations for DCIS and invasive breast cancers. However, we noted a stronger association between MD and breast cancer for invasive versus DCIS cancers among women aged 55 to 64 years and ≥65 years. Three prior studies showed either similar associations of MD with DCIS [20,48] and invasive breast cancer or somewhat stronger association for invasive tumors [21], while Yaghjyan and colleagues reported a significantly stronger association for *in situ* versus invasive tumors among postmenopausal women in the NHS [15]. The current study had a greater number of DCIS cases (555 vs. ≤300) and stratified associations by three age groups. In fact, when we examined the association of MD and tumor type overall, we found no difference in the association by DCIS versus invasive cancer, underscoring the importance of examining age-specific associations.

Our findings of stronger associations between MD and large compared with small tumors within each age group are consistent with most prior studies [7-9,15,22]. In addition, we noted a stronger association between MD and tumors with lymph node involvement compared with node-negative tumors. Both of these associations are consistent with studies that have reported positive associations with MD and breast cancers with aggressive tumor characteristics, including positive lymph nodes and advanced stage [7-9,49]. However, we found no evidence of differential associations by tumor grade. Observations of stronger associations between MD and more advanced tumors (for example, large size, node-positive) could perhaps reflect a possible masking effect because of reduced mammographic sensitivity and consequent delay in diagnosis [20,22,23]. The mean time between mammography and diagnosis was 4.6 years, and many of these women were screened annually, reducing the possible influence of a masking effect. While we observed a slightly stronger association between MD and lobular (vs. ductal) breast tumors among all women, this was not as apparent in age-stratified analyses and is in contrast to previous studies, which reported similar associations by histology [10,12,18].

Taken together, our findings suggest that MD is a risk factor for all types of breast cancer. However, some differences in magnitudes of risk associated with MD were noted for specific tumor characteristics primarily related to detection (for example, tumor size and nodal involvement in women of all ages) and for tumor characteristics primarily related to tumor biology (tumor type in older women and ER status in younger women). These observations point to possible heterogeneity by tumor characteristics and, if confirmed and corroborated by molecular-based studies, provide insight into the biological mechanisms by which MD differentially influences cancer risk.

Some limitations to this study are worth noting. We pooled data from six different studies, which varied in study design, population characteristics, geography, and calendar year. Our study used clinical pathology information and not centralized pathology review. In addition, there may have been changes in diagnostic criteria over time (for example, differences in threshold for positivity for ER status or HER2 testing). For example, some of the earliest diagnoses from the NHS included in this analysis were from the early 1990s before fluorescent in situ hybridization testing was routine. However, we found no evidence of between-study heterogeneity in our results. Further, any differences would be expected to attenuate associations with MD. The majority of women in our study were Caucasian; therefore, our findings may not be generalizable to non-Caucasians. Finally, we lacked data on the detection method (for example, screening-detected vs. interval-detected cancer), which could have helped to clarify the extent to which our observations reflect delays in diagnosis or tumor biology.

Strengths of the study included the sample of over 3,400 breast cancer cases, providing sufficient statistical power to examine associations by age group; cases and controls (except MCBCS) selected from underlying prospective cohort studies or registries with a mean time between mammogram and breast cancer diagnosis of 4.6 years; and comparable estimates of percent MD across readers at the three study sites with demonstrated high intra-reader reliability (intraclass correlation = 0.91 to 0.97) and standardization of percent MD for pooled analyses. The lack of between-study heterogeneity further suggests that pooling these data is appropriate. Also, we had detailed information on tumor characteristics from pathology reports supplemented with information from tissue microarrays. Finally, all mammograms were screening in nature.

## Conclusion

We observed significant positive associations between MD and breast cancer risk across tumor characteristics and age groups. In addition, we noted stronger associations for tumors of large size and positive lymph nodes across all ages, and ER-negative status among women aged <55 years, suggesting that high MD may play an important role in tumor aggressiveness, especially in younger women. Understanding similarities and differences in etiologic pathways according to breast cancer tumor characteristics and age has important implications for development of risk models and tailored primary prevention. Incorporating MD and traditional breast cancer risk factors into risk prediction models overall and into models unique to subtype may improve their predictive ability and allow clinicians to identify women at increased risk of breast cancer for targeted prevention efforts. Moreover, because MD itself is a potentially modifiable risk factor [50], understanding how it is differentially associated with breast cancer subtypes by age, particularly subtypes with poor prognosis more commonly diagnosed in younger women, could lead to novel strategies to reduce breast cancer incidence.

## Abbreviations

DCIS: Ductal carcinoma *in situ*; ER: Estrogen receptor; HER2: Human epidermal growth factor receptor 2; MCBCS: Mayo Clinic Breast Cancer Study; MCMAM: Mayo Clinic Mammography Study; MD: Mammographic density; MMHS: Mayo Mammography Health Study; NHS: Nurses’ Health Study; OR: Odds ratios; PR: Progesterone receptor; SFMR: San Francisco Bay Area Breast Cancer SPORE and San Francisco Mammography Registry; UCSF: University of California, San Francisco.

## Competing interests

The authors declare that they have no competing interests.

## Authors’ contributions

CMV, KK, and RMT conceived of and designed the study, directed statistical analyses, interpreted results, substantially revised initial drafts of the paper and provided final review and approval. JS led all aspects pertaining to mammogram acquisition and measurement of mammographic density for UCSF; he also provided substantial contribution to the interpretation of results and final approval of the manuscript. AN was the project manager of the overall study, providing study coordination and data acquisition for all combined information, harmonization of all data elements, as well as providing substantive comments to the drafts of the paper as well as final approval. BF acquired images and estimated mammographic density on UCSF mammograms, reviewed the initial and final drafts and provided final approval. Y-YC, DV, and AHB provided substantive pathology expertise to inform tumor characteristics and combination of information across studies, reviewed the drafts and provided comments and final approval. LM, CGS, MRJ, and VSP performed statistical analyses and interpretation of data, with input from KAB, RMT, KK and CMV. FC is the principal investigator of the MCBCS study and contributed substantive data, interpretation, drafting the manuscript and final approval. SRC is the principal investigator of one of the UCSF SFMR sites and contributed substantive data, interpretation, drafting the manuscript and final approval. KAB contributed to interpretation of analyses, wrote the first draft of the manuscript, which was critically revised by RMT, KK, and CMV, and approved the final draft. All authors read and approved the final manuscript.

## Supplementary Material

Additional file 1 Additional methods, presenting detailed description of study populations and methods to standardize MD measurements. Click here for file

Additional file 2: Table S1 Presenting associations of MD with breast cancer by study (OR (95% confidence interval)); **Table S2.** Presenting pooled associations of categorical MD with breast cancer overall and by tumor type and age; and **Table S3.** Presenting pooled associations of categorical percent MD for morphological subtypes of invasive breast cancer by age. Click here for file
