# Supplementary material for: Mammographic density and risk of breast cancer by age and tumor characteristics
Source: Breast Cancer Res. 2013 Nov 4;15(6):R104. doi: 10.1186/bcr3570 (PMC3978749; doi:10.1186/bcr3570)
Supplement: Additional file 1 — Additional methods, presenting detailed description of study populations and methods to standardize MD measurements. [file bcr3570-S1.pdf]

Additional methods

Study populations

The participating studies included the Mayo Mammography Health Study (MMHS), Mayo Clinic Breast Cancer Study (MCBCS), Nurses' Health Study (NHS) and NHSII, Mayo Clinic Mammography Study (MCMAM), and the Bay Area Breast Cancer SPORE and San Francisco Mammography Registry (SFMR).

MMHS

The MMHS is a large, prospective mammography cohort study at the Mayo Clinic. Over three years (2003-2006), 19,948 women from the tri-state region (Minnesota, Wisconsin, Iowa) who were age 35 or older and had a screening mammogram at the Mayo Clinic were enrolled[1, 2]. On all participants, risk factor information was ascertained on a baseline questionnaire; serial mammogram films and blood samples were obtained from a large proportion of the cohort. Participants are passively followed for breast cancer incidence and mortality through linkage to clinic (Mayo Tumor Registry) and state cancer registries of MN, IA and WI and state death certificates. For the present analysis, a nested case-control design was used. Controls were matched to cases on age, menopausal status, year of exam, and state. MMHS contributed 404 cases and 1207 controls to the current analysis.

MCBCS

The MCBCS is an ongoing clinic-based breast cancer case-control study initiated in 2001 at the Mayo Clinic as described previously[3, 4]. Patients with breast cancer were recruited Minnesota, Iowa, Wisconsin, North Dakota, South Dakota, and Illinois. Controls were recruited from the general internal medicine practices at the Mayo Clinic and frequency-matched to cases

on age (5-year category), race, and state of residence. The analyses here are based on 261 cases and 179 controls from the MCBCS who had available mammograms at least six months prior to breast cancer.

Nurses' health studies

The NHS is a prospective cohort that was established in 1976 and follows 121,701 registered female nurses in the United States, aged 30–55 years at enrollment. Similarly, the NHSII is an ongoing prospective cohort study of 116,678 premenopausal women who were aged 25 to 42 at baseline in 1989. Self-administered questionnaires are collected every two years to update information on diseases and risk factors such as weight, family history of breast cancer, parity, use of oral contraceptives or postmenopausal hormones, and diet. Incident breast cancer diagnoses were identified by self-report on each biennial questionnaire and confirmed by medical record review. Nested case-control studies of breast cancer were established within sub-cohorts of NHS and NHSII participants who gave blood samples to investigate a wide range of biomarkers as potential predictors of breast cancer, as previously described[5-7]. In addition, screening mammograms performed close to the date of blood draw were collected for the majority of these women. Women with any type of cancer (other than non-melanoma skin cancer) at the time of the selection or dates of diagnosis prior to date of mammogram were excluded. This analysis includes 1108 cases and 2163 controls from NHS and 365 cases and 992 controls from NHSII.

MCMAM

The MCMAM is a retrospective breast cancer case–control study nested within the Mayo Clinic mammography screening practice in Rochester, Minnesota[8]. Subjects had a screening mammogram between 1997 and 2001, were aged 50 years or older, lived within a 120-mile

radius of Rochester, and were required to have had at least two screening mammograms two years prior to their breast cancer diagnosis (case subjects) or screening examination (control subjects). Control subjects (i.e., women without breast cancer) were matched to each case subject on age (within 5 years), date of screening examination (within 4 months), menopausal status, interval between first and last mammograms (within 8 months), number of previous screening mammograms (within 1 mammogram), and county of residence. All data were obtained from retrospective chart review. MCMAM contributed 372 cases and 679 controls to the current analysis.

SFMR

The SFMR is an ethnically and economically diverse population-based mammography registry whose overall goal is to collect demographic, clinical, and risk factor information, as well as mammographic interpretations, and cancer outcomes through linkage with state-wide, California population-based Surveillance, Epidemiology, and End Results (SEER) program[9-11]. The SFMR began prospective data collection on women undergoing mammography in San Francisco County in 1995 and expanded data collection to Marin County mammography facilities in 2003. Since the registry's inception, information on over 2 million mammography examinations and 30,101 breast cancers has been collected from 20 facilities that offer breast imaging. Two of the larger facilities that participate in the SFMR, UCSF and California Pacific Medical Center, serve as the underlying screening mammography cohort and contributed 904 cases and 1979 matched controls for this study.

Standardization of mammographic density measurements

Because of known differences in the distribution of percent mammographic density (MD) across different readers[12, 13], we standardized the percent ~~mammographic density (~~MD~~)~~ reads

made at different research centers or at different times for studies within research centers. These standardized values made it possible to perform analyses of percent MD that combined information across multiple groups and/or time points. An added benefit to this approach is that it can be easily applied to data from studies which are not included in the current report, making it possible to standardize MD measurements obtained from almost any dataset with MD measures using the similar computer-assisted thresholding methods.

For these analyses, we included data from three different sites, comprising 6 different studies. Differences in percent MD, as well as age, were observed between these studies. Supplementary Figure 1 highlights both the age effect on MD as well as the differences in the distribution of percent MD across the studies observed for breast cancer cases. We also noted that variance in MD differed across studies. A similar pattern was observed for control patients (not shown).

Additional Figure 1:

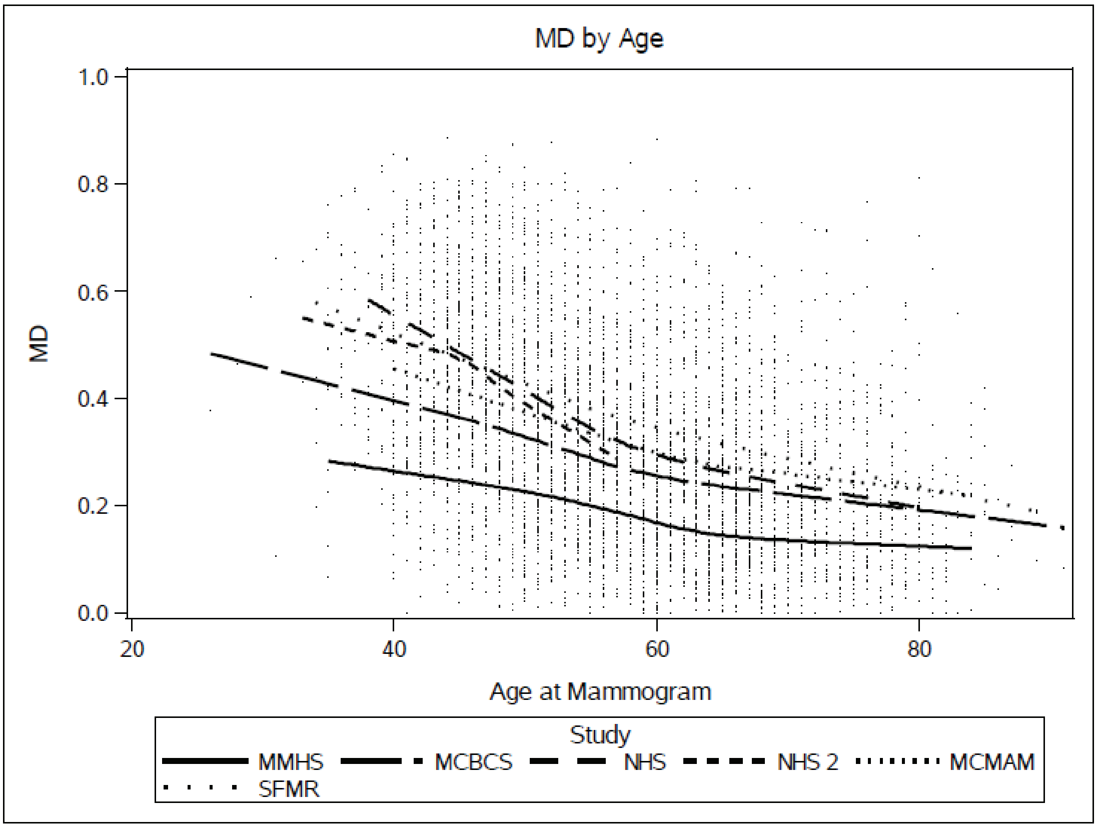

We used the age-dependent distribution of MD to standardize measures of MD obtained from groups of women of different ages. First, we modeled the age trend linearly and stabilized the residual variance by transforming the MD measures to the logit scale (logit of MD, or LMD). Because values of 0 are not permissible in the logit transformation, MD values of 0 were re-set to be one-half of the smallest non-zero value observed in each study. When plotted by age, the logit transformation showed age trends that were nearly linear. Second, the observed relationship between age and LMD was used as a framework to standardize MD across all study groups, as outlined in steps 1-6 below.

1) Quantile regression was performed to estimate the common linear trend in median LMD as a function of age, combining data from controls across all studies while allowing for different

intercepts by study. From this regression, the intercept corresponding to the study with the largest sample size was extracted and the estimate of age slope was obtained. These estimates are used as a reference age trend line for LMD within each study.

2) Quantile regression was performed within each study to estimate the linear trend in median LMD within the controls as a function of age. This analysis estimates the observed age trend in LMD for each study, and the residuals from the analysis give a de-trended per-person percent LMD value for each woman.

3) The estimates from these study-specific models were used to compute a predicted residual for each case. These predicted residuals were used to reflect the de-trended LMD value for each woman affected with breast cancer in the same way as the controls. This approach was applied in order to ensure that case-control differences in MD were maintained after applying the standardization.

4) We re-scaled the variances in LMD across study groups by computing the inter-quartile range (IQR) of the residuals from the controls, and dividing the residuals from steps 2 and 3 (cases and controls) by the study specific IQR. This produced study-specific residual distributions whose variances were scaled to the IQR of the controls in the appropriate study. Steps 2 through 4 result in a set of residuals that are re-centered (while accounting for age differences) and re-scaled such that they share roughly the same center (age-adjusted median

roughly equal to zero) and spread (age-adjusted variance scaled relative to the study-specific IQR).

5) The per-study residuals were multiplied by the IQR observed in the selected reference study. This step has the effect of re-scaling the residuals in such a way that the within-study variances are all roughly equal to one another.

6) We used the age at mammogram of each woman and applied the reference regression trend line from Step 1 to estimate the expected age-dependent LMD value for each woman. We then added the de-trended residual values available at the end of Step 5 to obtain the standardized LMD measurement for each woman.

The steps outlined above produce a measurement of LMD that retains the rank ordering of the within-study measurements while re-centering and rescaling their values such that the age by LMD relationship is roughly equivalent in the location of the trend and in the variability about the trend. Finally, the standardized percent MD is computed by back-transforming to the original scale as the inverse logit of the standardized LMD value. Supplementary Figure 2 shows the standardized percent MD measurements obtained when this method is applied to the data from six studies reported in this manuscript. The study-specific trend lines are now centered at approximately the same age trend, and the variability within study is more similar than it was previously.

Additional Figure 2:

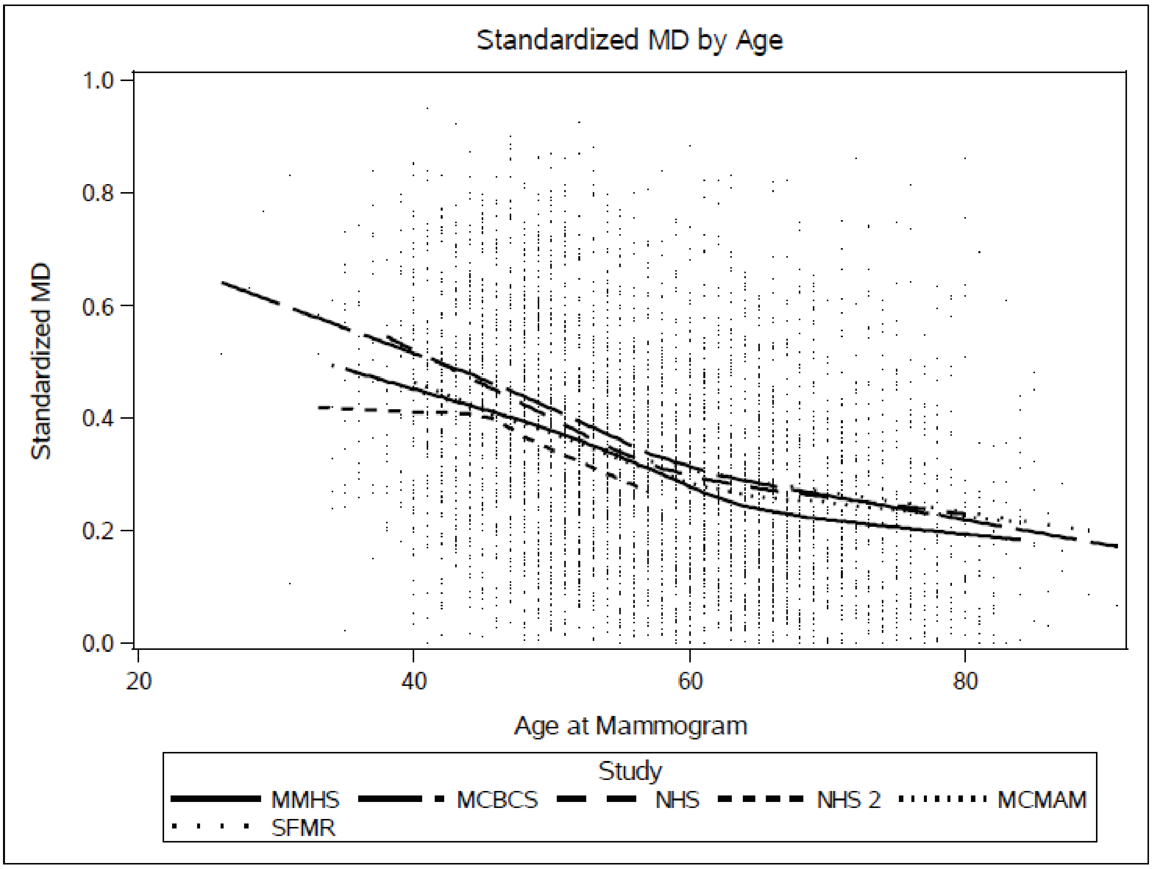

This standardization method makes it possible to rescale and re-center percent MD estimates in a way that the resulting percent MD estimates are approximately equivalent across studies that individually read MD using Cumulus. The use of standardized values in the context of epidemiologic analysis effectively reduces measurement error due to random variability in MD measurements across mammogram readers and study sites (e.g., "batch-to-batch" variability). From a practical standpoint, if data from a new study become available, the same steps can be followed to standardize the data to the measurements obtained from the reference study.

References

1. Heine JJ, Scott CG, Sellers TA, Brandt KR, Serie DJ, Wu FF, Morton MJ, Schueler BA, Couch FJ, Olson JE *et al*: **A novel automated mammographic density measure and breast * cancer risk**. *J Natl Cancer Inst* 2012, **104**(13):1028-1037.
2. Olson JE, Sellers TA, Scott CG, Schueler BA, Brandt KR, Serie DJ, Jensen MR, Wu FF, Morton MJ, Heine JJ *et al*: **The influence of mammogram acquisition on the mammographic density and breast cancer association in the Mayo Mammography Health Study Cohort**. *Breast Cancer Res* 2012, **14**(6):R147.
3. Kelemen LE, Couch FJ, Ahmed S, Dunning AM, Pharoah PD, Easton DF, Fredericksen ZS, Vierkant RA, Pankratz VS, Goode EL *et al*: **Genetic variation in stromal proteins decorin and lumican with breast cancer: investigations in two case-control studies**. *Breast Cancer Res* 2008, **10**(6):R98.
4. Wang X, Goode EL, Fredericksen ZS, Vierkant RA, Pankratz VS, Liu-Mares W, Rider DN, Vachon CM, Cerhan JR, Olson JE *et al*: **Association of genetic variation in genes implicated in the beta-catenin destruction complex with risk of breast cancer**. *Cancer Epidemiol Biomarkers Prev* 2008, **17**(8):2101-2108.
5. Tamimi RM, Hankinson SE, Colditz GA, Byrne C: **Endogenous sex hormone levels and mammographic density among postmenopausal women**. *Cancer Epidemiol Biomarkers Prev* 2005, **14**(11 Pt 1):2641-2647.
6. Tworoger SS, Sluss P, Hankinson SE: **Association between plasma prolactin concentrations and risk of breast cancer among predominately premenopausal women**. *Cancer Res* 2006, **66**(4):2476-2482.

7. Colditz GA, Hankinson SE: **The Nurses' Health Study: lifestyle and health among women**. *Nat Rev Cancer* 2005, **5**(5):388-396.

8. Vachon CM, Brandt KR, Ghosh K, Scott CG, Maloney SD, Carston MJ, Pankratz VS, Sellers TA: **Mammographic breast density as a general marker of breast cancer risk**. *Cancer Epidemiol Biomarkers Prev* 2007, **16**(1):43-49.

9. Kerlikowske K, Carney PA, Geller B, Mandelson MT, Taplin SH, Malvin K, Ernster V, Urban N, Cutter G, Rosenberg R *et al*: **Performance of screening mammography among women with and without a first-degree relative with breast cancer**. *Ann Intern Med* 2000, **133**(11):855-863.

10. Kerlikowske K, Shepherd J, Creasman J, Tice JA, Ziv E, Cummings SR: **Are breast density and bone mineral density independent risk factors for breast cancer?** *J Natl Cancer Inst* 2005, **97**(5):368-374.

11. Ziv E, Tice J, Smith-Bindman R, Shepherd J, Cummings S, Kerlikowske K: **Mammographic density and estrogen receptor status of breast cancer**. *Cancer Epidemiol Biomarkers Prev* 2004, **13**(12):2090-2095.

12. Yaffe MJ: **Mammographic density. Measurement of mammographic density**. *Breast Cancer Res* 2008, **10**(3):209.

13. Prevrhal S, Shepherd JA, Smith-Bindman R, Cummings SR, Kerlikowske K: **Accuracy of mammographic breast density analysis: results of formal operator training**. *Cancer Epidemiol Biomarkers Prev* 2002, **11**(11):1389-1393.
