# Supplementary material for: Mammographic density and risk of breast cancer by age and tumor characteristics
Source: Breast Cancer Res. 2013 Nov 4;15(6):R104. doi: 10.1186/bcr3570 (PMC3978749; doi:10.1186/bcr3570)
Supplement: Additional file 2: Table S1 — Presenting associations of MD with breast cancer by study (OR (95% confidence interval)); Table S2. Presenting pooled associations of categorical MD with breast cancer overall and by tumor type and age; and Table S3. Presenting pooled associations of categorical percent MD for morphological subtypes of invasive breast cancer by age. [file bcr3570-S2.pdf]

Additional Table ~~2~~ 1 Associations of mammographic density (MD) with breast cancer by study : odds ratios (95% confidence interval)[a]

**Category of MD**	**MMHS**	**MCBCS**	**NHS**	**NHS 2**	**MCMAM**	**SFMR**
0-10%	0.63 (0.45, 0.90)	0.72 (0.38, 1.35)	0.64 (0.50, 0.81)	0.46 (0.27, 0.79)	0.47 (0.32, 0.71)	0.78 (0.59, 1.03)
11-25% (ref)	1.00 (REF)	1.00 (REF)	1.00 (REF)	1.00 (REF)	1.00 (REF)	1.00 (REF)
26-50%	1.67 (1.26, 2.22)	1.96 (1.16, 3.34)	1.57 (1.31, 1.90)	1.38 (1.00, 1.92)	1.70 (1.22, 2.35)	1.78 (1.43, 2.21)
51%+	1.70 (1.11, 2.59)	2.95 (1.48, 5.87)	2.09 (1.65, 2.64)	1.67 (1.15, 2.42)	2.40 (1.51, 3.81)	2.43 (1.88, 3.14)

[a] Adjusted for age and body mass index.

Additional Table ~~3~~ 2 Pooled associations of categorical MD[a] with breast cancer overall and by tumor type and age

	Age < 55			Age 56-64			Age ≥ 65			
	Cases	Controls	OR[b] (95% CI) [d]	Cases	Controls	OR[b] (95% CI) [cd]	Cases	Controls	OR[b] (95% CI)[cd]	$P_{age\text{-}interaction}$
All breast cancer										0.01
0-10%	88	452	0.56 (0.43, 0.74)	155	523	0.60 (0.48, 0.76)	187	510	0.67 (0.54, 0.85)	
11-25% (ref)	271	852	1.00 (REF)	292	690	1.00 (REF)	309	706	1.00 (REF)	
26-50%	665	1299	1.64 (1.38, 1.95)	430	629	1.78 (1.47, 2.16)	284	458	1.53 (1.24, 1.89)	
51%+	509	770	2.11 (1.74, 2.56)	144	189	2.16 (1.65, 2.83)	80	121	1.84 (1.32, 2.56)	
Tumor Type										
Invasive										
0-10%	71	452	0.54 (0.4, 0.73)	136	523	0.66 (0.51, 0.84)	168	510	0.68 (0.53, 0.86)	0.02
11-25% (ref)	225	852	1.00 (REF)	238	690	1.00 (REF)	267	706	1.00 (REF)	
26-50%	523	1299	1.57 (1.3, 1.89)	358	629	1.82 (1.48, 2.24)	251	458	1.58 (1.27, 1.97)	
51%+	408	770	2.08 (1.69, 2.55)	126	189	2.29 (1.73, 3.04)	74	121	1.99 (1.41, 2.8)	
In situ										
0-10%	15	452	0.65 (0.35, 1.19)	18	523	0.36 (0.2, 0.63)	19	510	0.63 (0.36, 1.11)	
11-25% (ref)	43	852	1.00 (REF)	54	690	1.00 (REF)	42	706	1.00 (REF)	
26-50%	136	1299	1.99 (1.38, 2.85)	71	629	1.59 (1.08, 2.34)	33	458	1.22 (0.75, 1.97)	
51%+	101	770	2.39 (1.61, 3.56)	17	189	1.47 (0.82, 2.66)	6	121	0.96 (0.39, 2.36)	
$P_{heterogeneity}$			0.68			0.15			0.42	

[a] MD, percent mammographic density;
[b] OR, odds ratio;
[c] CI, confidence interval.
[d] Adjusted for age, body mass index, and study.

Additional Table 4-3 Pooled associations of categorical percent mammographic density for morphological subtypes of invasive breast cancer, by age

	Age < 55			Age 55-64			Age ≥ 65			
	**No. cases**	**No. controls**	**OR[a] (95% CI)[bc]**	**No. cases**	**No. controls**	**OR[a] (95% CI)[bc]**	**No. cases**	**No. controls**	**OR[a] (95% CI)[bc]**	**$P_{age\text{-}interaction}$**
Histology[d]										0.15
Ductal										
0-10%	61	452	0.58 (0.42, 0.8)	103	523	0.66 (0.5, 0.87)	117	510	0.67 (0.51, 0.88)	
11-25% (ref)	178	852	1.0 (REF)	182	690	1.0 (REF)	196	706	1.0 (REF)	
26-50%	424	1299	1.61 (1.31, 1.97)	276	629	1.83 (1.46, 2.3)	184	458	1.58 (1.24, 2.03)	
51%+	325	770	2.11 (1.68, 2.64)	93	189	2.21 (1.62, 3.02)	48	121	1.77 (1.19, 2.62)	
Lobular										
0-10%	4	452	0.24 (0.08, 0.71)	19	523	0.64 (0.36, 1.16)	21	510	0.55 (0.32, 0.96)	
11-25% (ref)	25	852	1.0 (REF)	34	690	1.0 (REF)	41	706	1.0 (REF)	
26-50%	44	1299	1.22 (0.73, 2.04)	38	629	1.31 (0.8, 2.12)	37	458	1.56 (0.97, 2.5)	
51%+	50	770	2.47 (1.44, 4.24)	19	189	2.36 (1.29, 4.33)	15	121	2.9 (1.5, 5.61)	
$P_{heterogeneity}$[e]			0.12			0.45			0.39	
Histologic grade										0.21
Well differentiated										
0-10%	14	452	0.46 (0.25, 0.85)	33	523	0.49 (0.32, 0.76)	60	510	0.7 (0.49, 1)	
11-25% (ref)	54	852	1.00 (REF)	80	690	1.0 (REF)	94	706	1.0 (REF)	
26-50%	116	1299	1.42 (1.0, 2.0)	99	629	1.42 (1.03, 1.97)	80	458	1.4 (1.01, 1.95)	
51%+	108	770	2.32 (1.6, 3.35)	36	189	1.73 (1.11, 2.69)	25	121	1.92 (1.16, 3.19)	
Moderately differentiated										

0-10%	32	452	0.67 (0.43, 1.04)	48	523	0.6 (0.41, 0.87)	67	510	0.75 (0.53, 1.07)	
11-25% (ref)	82	852	1.00 (REF)	91	690	1.0 (REF)	97	706	1.0 (REF)	
26-50%	201	1299	1.66 (1.25, 2.19)	132	629	1.79 (1.33, 2.42)	98	458	1.68 (1.22, 2.3)	
51%+	151	770	2.14 (1.58, 2.92)	49	189	2.38 (1.59, 3.55)	33	121	2.4 (1.51, 3.83)	
Poorly differentiated	466									
0-10%	19	452	0.54 (0.31, 0.93)	33	523	0.99 (0.6, 1.63)	24	510	0.43 (0.26, 0.72)	
11-25% (ref)	58	852	1.00 (REF)	37	690	1.0 (REF)	53	706	1.0 (REF)	
26-50%	156	1299	1.8 (1.3, 2.48)	70	629	2.46 (1.6, 3.76)	48	458	1.58 (1.04, 2.41)	
51%+	109	770	2.1 (1.47, 3.02)	25	189	3.29 (1.89, 5.73)	9	121	1.31 (0.61, 2.79)	
$P_{heterogeneity}$[e]			0.54			0.88			0.83	
Tumor size										0.37
<1.1 cm										
0-10%	34	452	0.9 (0.59, 1.39)	63	523	0.79 (0.56, 1.11)	82	510	1.01 (0.72, 1.41)	
11-25% (ref)	74	852	1.00 (REF)	100	690	1.0 (REF)	91	706	1.0 (REF)	
26-50%	153	1299	1.3 (0.97, 1.75)	115	629	1.31 (0.97, 1.76)	85	458	1.55 (1.12, 2.15)	
51%+	117	770	1.61 (1.16, 2.24)	34	189	1.33 (0.86, 2.06)	21	121	1.57 (0.92, 2.67)	
1.1 - 2.0 cm										
0-10%	24	452	0.44 (0.27, 0.7)	45	523	0.6 (0.41, 0.89)	53	510	0.51 (0.35, 0.73)	
11-25% (ref)	95	852	1.00 (REF)	85	690	1.0 (REF)	114	706	1.0 (REF)	
26-50%	211	1299	1.49 (1.14, 1.94)	140	629	2.02 (1.5, 2.73)	104	458	1.5 (1.11, 2.04)	
51%+	166	770	1.98 (1.48, 2.66)	60	189	3.09 (2.1, 4.54)	28	121	1.68 (1.04, 2.72)	

2.1+ cm										
0-10%	11	452	0.31 (0.15, 0.6)	21	523	0.47 (0.27, 0.81)	25	510	0.41 (0.25, 0.68)	
11-25% (ref)	51	852	1.00 (REF)	47	690	1.0 (REF)	56	706	1.0 (REF)	
26-50%	139	1299	2 (1.42, 2.82)	89	629	2.49 (1.69, 3.66)	53	458	1.73 (1.15, 2.6)	
51%+	109	770	2.88 (1.97, 4.21)	27	189	2.92 (1.73, 4.93)	23	121	3.65 (2.08, 6.39)	
$P_{heterogeneity}$ [e]			<0.01			<0.01			<0.01	
Involvement of lymph nodes										0.24
Negative										
0-10%	49	452	0.54 (0.38, 0.76)	98	523	0.65 (0.49, 0.87)	112	510	0.72 (0.54, 0.95)	
11-25% (ref)	160	852	1.00 (REF)	174	690	1.0 (REF)	174	706	1.0 (REF)	
26-50%	334	1299	1.4 (1.13, 1.73)	249	629	1.72 (1.37, 2.16)	163	458	1.56 (1.21, 2.01)	
51%+	267	770	1.89 (1.49, 2.39)	89	189	2.19 (1.6, 3)	51	121	1.99 (1.35, 2.94)	
Positive										
0-10%	18	452	0.55 (0.31, 0.97)	27	523	0.64 (0.39, 1.06)	20	510	0.35 (0.21, 0.61)	
11-25% (ref)	51	852	1.00 (REF)	50	690	1.0 (REF)	56	706	1.0 (REF)	
26-50%	158	1299	2.15 (1.53, 3.02)	85	629	2.1 (1.44, 3.06)	56	458	1.78 (1.19, 2.67)	26-50%
51%+	120	770	2.86 (1.98, 4.14)	30	189	2.61 (1.58, 4.3)	16	121	2.32 (1.25, 4.31)	
$P_{heterogeneity}$ [e]			0.11			0.75			0.04	
ER status										0.04
Negative										
0-10%	8	452	0.35 (0.16, 0.76)	23	523	0.86 (0.49, 1.49)	17	510	0.53 (0.29, 0.98)	
11-25% (ref)	36	852	1.00	35	690	1.0	35	706	1.0	

			(REF)			(REF)			(REF)	
26-50%	104	1299	2.08 (1.4, 3.11)	64	629	2.03 (1.31, 3.14)	24	458	1.11 (0.64, 1.91)	
51%+	80	770	2.84 (1.83, 4.4)	17	189	1.84 (0.99, 3.41)	6	121	1.1 (0.44, 2.75)	
Positive										
0-10%	60	452	0.57 (0.42, 0.79)	104	523	0.61 (0.46, 0.8)	143	510	0.69 (0.53, 0.89)	
11-25% (ref)	182	852	1.00 (REF)	192	690	1.0 (REF)	222	706	1.0 (REF)	
26-50%	392	1299	1.44 (1.17, 1.76)	276	629	1.78 (1.42, 2.22)	215	458	1.64 (1.3, 2.07)	
51%+	315	770	1.96 (1.56, 2.45)	102	189	2.37 (1.75, 3.21)	64	121	2.1 (1.46, 3.02)	
$P_{heterogeneity}^{e}$			0.09			0.38			0.39	
PR status										0.10
Negative										
0-10%	18	452	0.48 (0.28, 0.84)	42	523	0.79 (0.52, 1.2)	31	510	0.59 (0.37, 0.94)	
11-25% (ref)	63	852	1.00 (REF)	67	690	1.0 (REF)	57	706	1.0 (REF)	
26-50%	144	1299	1.59 (1.15, 2.18)	90	629	1.55 (1.1, 2.18)	54	458	1.6 (1.07, 2.39)	
51%+	104	770	1.96 (1.38, 2.8)	33	189	2.01 (1.26, 3.2)	15	121	1.9 (1.02, 3.55)	
Positive										
0-10%	51	452	0.58 (0.41, 0.81)	86	523	0.59 (0.44, 0.79)	130	510	0.7 (0.54, 0.92)	
11-25% (ref)	152	852	1.00 (REF)	161	690	1.0 (REF)	199	706	1.0 (REF)	
26-50%	354	1299	1.56 (1.26, 1.94)	248	629	1.91 (1.51, 2.41)	183	458	1.55 (1.21, 1.97)	
51%+	287	770	2.16 (1.7, 2.74)	85	189	2.33 (1.69, 3.23)	54	121	1.95 (1.33, 2.86)	
$P_{heterogeneity}^{e}$			0.85			0.21			0.88	
HER2 status										0.03
Negative										
0-10%	40	452	0.52	49	523	0.5	101	510	0.83	

			(0.36, 0.77)			(0.35, 0.73)			(0.61, 1.13)
11-25% (ref)	129	852	1.00 (REF)	113	690	1.0 (REF)	125	706	1.0 (REF)
26-50%	288	1299	1.52 (1.2, 1.93)	149	629	1.69 (1.27, 2.25)	123	458	1.67 (1.25, 2.25)
51%+	217	770	1.94 (1.49, 2.52)	48	189	1.84 (1.23, 2.75)	34	121	1.9 (1.2, 3.01)
Positive									
0-10%	7	452	0.48 (0.2, 1.14)	11	523	0.9 (0.4, 2.02)	13	510	0.49 (0.24, 1.01)
11-25% (ref)	23	852	1.00 (REF)	15	690	1.0 (REF)	24	706	1.0 (REF)
26-50%	68	1299	2.03 (1.24, 3.34)	28	629	2.44 (1.26, 4.71)	20	458	1.41 (0.75, 2.64)
51%+	44	770	2.19 (1.26, 3.82)	9	189	2.64 (1.1, 6.36)	8	121	2.28 (0.96, 5.46)
$P_{heterogeneity}$[e]			0.63			0.57			0.48

[a] OR, odds ratio

[b] CI, confidence interval

[c] Adjusted for age and body mass index, and study

[d] Mixed and other histology categories are excluded

[e] Test of heterogeneity in associations by subtype (P_{trend} for categories with natural ordering, i.e., tumor size and histologic grade)
